# The resilience of nursing staffs in nursing homes: concept development applying a hybrid model

**DOI:** 10.1186/s12912-022-00913-2

**Published:** 2022-05-26

**Authors:** Sung Ok Chang, Eun Young Kim

**Affiliations:** 1 College of Nursing and BK21 FOUR R&E Center for Learning Health Systems, Korea University, Seoul, Republic of Korea; 2 College of Nursing, Korea University, 145, Anam-ro, Seongbuk-gu, 02841 Seoul, Republic of Korea

**Keywords:** Nursing homes, Nursing staff, Resilience, psychological, Qualitative research

## Abstract

**Background:**

The resilience of nursing home (NH) nursing staff is emphasized to improve the quality of care provided, but the concept has not been clearly defined. By composing such a definition through concept development, a basis for active research in the future can be established.

**Aim:**

To identify the definition and conceptual characteristics of the concept of resilience of NH nursing staffs.

**Method:**

In this study, the concept was developed using Schwartz-Barcott and Kim’s hybrid model, which included theoretical, fieldwork, and final analysis stages. In the theoretical stage, a literature review on the definition and measurement of concepts was performed. For the fieldwork stage, 22 interviews were conducted with 7 participants, and a content analysis was performed. During the final analysis stage, the results of the theoretical and field work stages were integrated.

**Results:**

Three dimensions, eight attributes, and three types were identified. The three dimensions are internal resources, external support, and positive coping with situations as they arise; the eight attributes are optimism, patience, mindfulness, supportive relationships, available resources, work-life boundary setting, self-development, and growth; and the three types are those who want to adapt themselves to the situation, those who actively seek to cope with stressful situations, and those who hold positive expectations for the future.

**Conclusion:**

Coping with difficult situations using internal resources and external support was a unique trait revealed in the resilience of NH nursing staff members. This study provided future research directions to improve the resilience of NH nursing staffs by revealing the characteristics of their resilience.

## Introduction

As the elderly population increases worldwide, interest in nursing homes (NHs) that can serve as living spaces for this growing cohort and provide them palliative care is increasing [1]. A NH is a facility that provides 24-hour support for their activities of daily living (ADL) and instrumental activities of daily living (IADL) for patients with physical and cognitive disabilities [2]. NHs reflect an aging society’s demand for supervised elderly care, leading to greater demands for quality nursing care and nursing competency in NHs [3]. In the United States, the highest proportion of nursing professionals working in NHs are registered nurses (RNs), licensed vocational nurses (LVNs), licensed practical nurses (LPNs), certified nursing assistants (CNAs), and physical therapists (PTs) [4]. In Korea, nurses, nursing assistants, and care workers account for the largest proportion of NH personnel. As such, it is necessary to pay attention to the job satisfaction and difficulties of such healthcare professionals because the operational purposes of a NH and its staff have unique characteristics that differentiate them from other institutions. Currently, the number of nursing personnel working in NHs is insufficient at around the world, often making one nurse responsible for the care of many elderly people in circumstances that vary from facility to facility [5, 6]. The recent pandemic is exacerbating this difficult situation [5]. Because of the abovementioned problems, nursing staffs in NHs show burnout, a high intention to resign, and low job satisfaction [7]. Such negative experiences of nurses not only lower the quality of nursing care they provide [8], but also lead to resignations, resulting in wasted social costs [9]. Because these problems eventually lead to a lower quality of care provided to patients [8], we need to examine the various difficulties experienced by nursing personnel in NHs and conduct research on protection strategies that can buffer them from the negative effects of their difficulties [10].

Resilience is an important concept for explaining protection strategies [10] and refers to the ability to adapt to physical and psychological requirements in difficult situations. Resilience is a positive concept that helps individuals maintain their psychological health [11] and is attracting attention as an important factor in reducing the burnout and psychological burden of nurses and for promoting their mental health [12, 13].

To date, resilience has been studied in various fields, including sociology, education, and psychology. In the field of nursing, because of the growing interest in nurses’ quality of life, research on nurses’ resilience has become more active [14]. In previous studies, the attributes of nurse’s resilience have been defined as a complex concept, one that includes self-efficacy, home, coping, social support, work-life balance, humor, optimism, and being realistic [13, 15]. Since nursing resilience has been found to be helpful in reducing burnout, stress, and the rates of retirement and resignation among nurses [16–18], researchers are making efforts to promote nurses’ resiliency in a variety of ways.

As asserted in the Ungar study [19, 20], resilience requires a context-sensitive approach, and to correctly identify attributes, an approach that fits the culture to which the concept belongs must be employed. NH nursing staff resilience is a concept that can improve the quality of life of nursing staff working in NHs and can provide practical help in solving one of the serious problems these facilities currently face, a shortage of nursing staff. Therefore, if additional studies prove the positive effects of resilience for NH nursing staff in the future, it will potentially have a positive effect on the social issues of elderly care through informing policy-level discussions. Therefore, it is necessary to study the concept of resilience for nursing staff working in an environment with unique characteristics, such as a NH to use such studies as a basis for further research. Since few studies on the resilience of NH nursing staff that have been conducted and since it is a concept based on the perceptions formed in the clinical field, it is essential to reflect those clinical perceptions. A hybrid model of concept development includes both theoretical and empirical processes and is useful for studying meaningful and central conceptual phenomena in nursing [21]. Therefore, this study was conducted to explore the essence of the concept of the resilience of nurses in NHs using a hybrid model.

## Aim

The purpose of this study was to identify the definition and conceptual characteristics of the concept of resilience for NH nursing staff members through theoretical, fieldwork, and final analysis stages.

## Methods

This study’s aim is to develop a concept of resilience in regard to NH nursing personnel using Schwartz-Barcott and Kim’s hybrid model [22]. This model [22] is an approach used for conceptual analysis and includes both theoretical and empirical processes. Because nursing is based on the perceptions formed in clinical practice, such a hybrid model is a useful method to study meaningful and central phenomena in nursing [21]. It consists of three phases: a theoretical phase, a fieldwork phase, and a final analytical phase. Therefore, in this study, first, through a literature review in the theoretical stage, we examined what the properties of NH nursing staff resilience are, how they are measured, and how they relate to similar concepts. Then, during the fieldwork stage, participants who could explain the concept were selected, and how they experienced the resilience shown in the theoretical stage and what characteristics the concept presented in practice were analyzed. In the final analysis stage, the properties of resilience of NH nursing staff personnel were identified and defined by comprehensively analyzing the theoretical and fieldwork stages.

### Theoretical stage

**Fig. 1:**
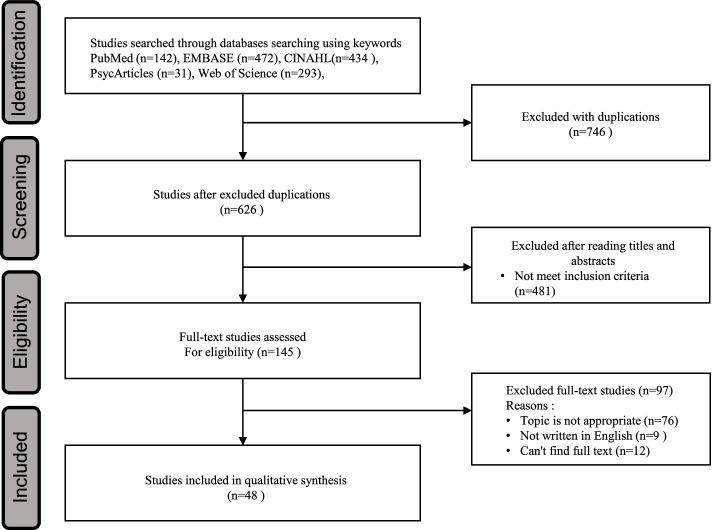
The overall flowchart of the literature review

A literature review focusing on the conceptual definition and measurements of resilience of NH nursing staff was undertaken. The overall flow chart of the literature review is presented in Fig. 1.

The search was conducted based on the following questions: “What is the essence of the concept?”, “How can the essence of the concept be clearly defined?”, and “How can the concept be fleshed out to improve measurability?” First, to examine the concept’s essential meaning, its dictionary meaning was considered. Second, a literature search was conducted in December 2021. A search of the literature concerning the concept was performed using the terms “resilience,” “nursing home,” “nursing staff” using the databases of PubMed, EMBASE, CINHAL, Web of Science, and PsychyINFO. Since the literature on the resilience of nursing staff in NHs was found to be insufficient, we performed a comprehensive analysis by including the literature on general resilience in the theoretical stage. A literature search was performed by selecting a search term representing the above search terms from the index list of each database. The inclusion criteria for the literature search were (a) studies aimed at exploring resilience, (b) studies in which the keywords used in the search were used in titles and abstracts, (c) publications in English, and (d) full-text search availability. The exclusion criteria were studies for which resilience-related data did not includ. The literature types included in the review were qualitative research, quantitative research, mixed method research, meta-synthesis, meta-analysis, and instrument development research. There was no restriction on publication year, and the searched literature was managed using the EndNote program. The literature review was conducted with a focus on the definition and measurement of concepts and sought to capture the extremes of conceptualization and the use of selected concepts across fields. The literature search using 5 databases found 1372 studies, 746 of which were excluded due to duplication. Of the 626 studies, 481 were excluded for not meeting the inclusion criteria, and the full texts of the remaining 145 studies were checked. After checking their full texts, 97 studies were excluded as inappropriate, leaving the final 48 studies that were selected for the literature review. The literature search was performed by the first author, and the literature was selected through discussion with the second author at each stage of the exclusion process.

The two researchers repeatedly read the selected literature and derived the attributes of resilience. By comparing the derived attributes, consensus was reached through discussions about any differences in results, and the attributes were classified into dimensions through the process of classifying similar viewpoints. The literature measuring resilience went through a separate analysis process that focused on which indicator was used to measure resilience.

### Fieldwork stage

The fieldwork stage aims to refine a concept by integrating and extending the analysis initiated in the theoretical stage with ongoing empirical observations [22]. When selecting a setting and participants, the possibility of frequently observing the phenomenon to be studied, whether participatory observation is possible, and the possibility that the researcher can conduct and continue participatory observation in the setting should be considered [22]. Therefore, the researchers judged that NHs were a suitable environment for studying this research concept, and three NHs in Korea were selected as the setting. After in-depth discussions with the NH managers, participants who potentially embodied the attributes of resilience revealed in the theoretical stage were recommended by the managers. In the fieldwork stage of the hybrid model, conducting repeated interviews with three to six participants is suggested.

In order to lower the risk of potential trauma among participants, the researchers followed the recommendations of Disaster Mental Health Research Ethics to recruit and interview participants [23]. Before conducting the study, a sufficient explanation of the study was provided to the subjects, and only those who voluntarily expressed their intention to participate were selected and informed consent was obtained. It was fully explained that the data collected for the study were anonymized and secure, and that the study could be stopped at any time if desired. Seven staff members who voluntarily expressed their intention to participate in the study were selected as participants.

The interviews were conducted by the first and corresponding authors. The hybrid model’s recommended setting is a place where observation is possible, one where the researcher can engage in participatory observation and where participant interviews can be held. However, due to the recent COVID-19 pandemic, participatory observation was impossible, and in-depth interviews were conducted using video conferencing (Zoom) and phone calls rather than on site. Information on the situations and atmosphere of the clinical settings, which could not be collected through observation, was collected through questions. The interviews, recorded with the consent of the participants, were conducted individually from December 20, 2021, to January 20, 2022. Each participant was interviewed three to five times for 50 to 90 minutes. Interviews were conducted until saturation was reached when no new content was confirmed, and the total number of interviews was 22. The interview questions were structured based on the findings of the theoretical stage, and additional questions were asked based on the answers to the following questions: “Is there evidence of the resilience of nursing staff in this work environment?”, “If so, to what extent can the existing definitions fully grasp the essence of this phenomenon?”, “What is the best indicator of resilience?”, and “Please share your experiences of overcoming the adversity you experienced at the nursing home.” The interviews were conducted in a natural conversational format so that the concept’s attributes could be well expressed. Interviews were conducted until the subject was deemed saturated with no additional new content, while additional interviews were conducted if any parts of the interviews required additional explanation. The interview data analysis was conducted using the content analysis method [24].

The hybrid model can use Wilson’s [25] typology to clarify the essential characteristics of a concept. Since the participants selected in this study clearly showed the attributes of resilience, this study only presented model cases that well reflect the concept.

Researchers took time to reflect upon themselves to guard against any prejudices and biases about the experience of NH nursing staff resilience. The two researchers completed each process over a substantial period of time, and the reliability of the results was ensured by confirming the valid interpretation and classification of the interview data [26]. The researchers confirmed the true value of the data by summarizing the interview and confirming the participants’ points of view with the data.

### Final analytic stage

The final analytic stage clarifies the concept by reviewing the results of the data collected with the insights gained in the theoretical and fieldwork stages. The researcher then takes a step back from the details and intensity of the fieldwork stage and reexamines the results, focusing on the initial interest in the topic. In the final analytic stage, the results from the theoretical and the fieldwork stages are compared, and a definition that could support both the literature and the participant’s’ points of view are refined and generated. We checked whether the meaning extracted from the theoretical phase was confirmed in the fieldwork phase, what attributes were in the theoretical phase but could not be confirmed in the fieldwork phase, and what attributes were not in the theoretical phase but were confirmed in the fieldwork phase. Through this process, the concept was redefined after revision.

In addition, the strength of the indicator characteristics that appeared through the literature review was analyzed for each participant, and the type was derived by classifying it based on the strength of the indicator characteristics that emerged.

#### Ethical considerations

This study was approved by the Korea University Institutional Review Board (KUIRB-2021-0259-02). Before conducting the interviews, the researcher explained the purpose and method of the study to each participant, explained that all data would be used only for the study, and obtained their voluntary consent. Participants were informed that their personal information would be thoroughly protected and that it would be disposed of after a certain period after its analysis.

## Results

### Theoretical stage

#### Dictionary meaning of the resilience of nursing staff in nursing homes

According to the American Heritage Dictionary [27], resilience is defined as “The ability to recover quickly from illness, change, or misfortune, buoyancy. The property of a material that enables it to resume its original shape or position after being bent, stretched, or compressed; elasticity.” As for NHs, they are defined as “a private establishment that provides living quarters and care for chronically ill, usually elderly patients” [27]. Nursing staff refers to personnel who provide nursing care in NHs and in South Korea is composed of nurses, nursing assistants, and care workers [28, 29]. Thus, the resilience of NH nursing staff can be defined as the ability and process by which NH nursing staff quickly recover from the negative experiences experienced while working in a NH and return to their daily activities and work duties.

#### Dimensions, attributes, and measurements in the theoretical stage

***Dimensions*** In the theoretical stage, the concept’s dimensions were confirmed through a literature analysis as “personal adversity management ability,” “a dynamic process of positively adapting through adversity,” and “maintaining psychological/social health.”

- **Personal adversity management ability:** In this dimension, resilience is regarded as an individual’s unique characteristics and abilities in the process of coping with adversities. Resilience has been described as the ability to achieve successful adaptation in the face of high-risk conditions, chronic stress, or subsequent trauma [30], as well as the ability to sustain a functional life [31]. Werner and Smith [32] defined it as the ability to recover from adversity, while Conner and Davidson [33] defined it as the ability to successfully cope with stress and adversity. Resilience has also been described as the ability to find psychological stability and balance in the face of severe stress or unavoidable difficulties [34], the ability to achieve successful adaptation in the face of danger, stress, and trauma [35], and the effort to restore or maintain personal balance and mental and physical health. A comprehensive examination of the above definitions finds that this dimension emphasizes personal competence, strength, and the ability to overcome stress or trauma and achieve successful adaptation to difficult situations.
- **A dynamic process of positively adapting through adversity:** Margalit [35] defined resilience as a dynamic process of positive adaptation experienced through adversity, while Windle [36] defined it as a process of effectively regulating, adapting, and managing the underlying factors of stress and trauma. Similarly, Dyer and Mcguinness [37] described it as the process by which humans deal with adversity and continue with their lives, and Garcia-Dia et al. [38] described it as a dynamic process of recovering from adversity. As such, resilience in this dimension refers to the series of processes involved in experiencing, recovering from, and adapting to adversity.
- **Maintaining psychological/social health**: Kander [34] defined resilience as finding psychological stability in the face of severe stress or unavoidable difficulties, and Bonanno [39] defined it as maintaining stable, continuous mental and physical health. This refers to maintaining one’s psychological and social health after the process of overcoming and adapting to the underlying factors of stress and trauma. Resilience in this dimension means ultimately overcoming stress and trauma, returning to daily life, and living a stable life.

***Attributes*** The attributes identified at the theoretical stage were adaptability, interpersonal relationships, flexibility, persistence, self-regulation, positivity, self-care, and self-esteem.

***Measurements*** The Connor-Davidson Resilience Scale [40] is a self-rated assessment that measures a person’s sense of control and tenacity, self-efficacy, tolerance of negative affect and ease of recovery, positive acceptance of change, and secure relations. Friborg et al.’s Resilience Scale for Adults [41] measures personal and social competence, family coherence, social support, and personal structure. The Wagnild & Young Resilience Scale [42], derived from a qualitative study of older women (aged 67–92), measures equality, perseverance, self-reliance, meaningfulness, and existential aloneness, while Rahman et al.’s Medical Professionals Resilience Scale [43] measures control, involvement, resourcefulness, and growth. From a holistic understanding of the above scales, when measuring resilience in nursing, self-belief, dependable relationships, resources for support, willingness to grow, management of stress, positivity, and flexible thinking were identified as indicators by which to measure resilience.

#### Working definition

The working definition of NH nursing staff resilience refers to the entire process of maintaining psychological/social health while undergoing a dynamic process of positively adapting through adversity with the personal crisis management ability to overcome crises in stressful or emergency situations.

### Fieldwork stage

The general characteristics of the participants are presented in Table 1. As a result of analyzing the collected interview data, the three dimensions of emotional stability, bonding with colleagues, and coping with situations, and the seven attributes of optimism, patience, mindfulness, empathy with colleagues, cooperation with colleagues, setting work boundaries, and self-development were derived.

**Table 1:** General characteristics of the participants (*N* = 7)

Characteristics	N (%)	Mean (SD)
Occupation		
Nurse	3 (42.86)	
Nursing assistant	2 (28.57)	
Care worker	2 (28.57)	
Age (years)		
30–39	1 (14.29)	52.14 (9.14)
40–49	1 (14.29)	
50–59	3 (42.86)	
60–69	2 (28.57)	
Work experiences in NH (mean, years)		
1–5	3 (42.86)	7.14 (8.05)
6–10	3 (42.86)	
10–15	1 (14.29)	
Gender		
Women	7 (100)	
Men	0	
Education		
College	5 (71.43)	
Master’s degree	2 (28.57)	

#### Emotional stability

This dimension refers to overcoming adversities through emotional stability. Three attributes are included in this dimension: optimism, patience, and mindfulness. This dimension centers on maintaining a positive perspective by acknowledging and accepting various situations through emotional stability.

*“I think everything is a matter of my heart (mindfulness). If I take good care of my mind, everything is supposed to work out (Optimism).” (Case 2)*

*“People these days think it's a problem because they're too impatient. Even if difficult things happen, you need to have a little patience (Patience).” (Case 4)*

#### Bonding with colleagues

In the second dimension, the bond with colleagues is the driving force for overcoming the crisis. This dimension includes the attributes of empathy and cooperation with colleagues.

*“Caring for a dementia patient is really hard. Only my colleagues can empathize with this situation (Empathy with colleagues).” (Case 6)*

*“When the workload is too much, the nurses or nursing assistants I work with help me, and I try to help them (Cooperation with colleagues).” (Case 7)*

#### Coping with various situations

The third dimension focuses on overcoming crises by actively responding to difficult situations. This dimension includes the attributes of work-life boundary setting, self-development, and growth.

*“The most important thing is to have personal time. I try to forget everything by getting away from work and getting enough rest, both physically and mentally (Work-life boundary setting).” (Case 3)*

*“Even in difficult situations, if I move forward toward the future instead of staying in the present, I can have vitality in my life (Self-development, Growth).” (Case 5)*

### Final analytical stage

**Fig. 2:**
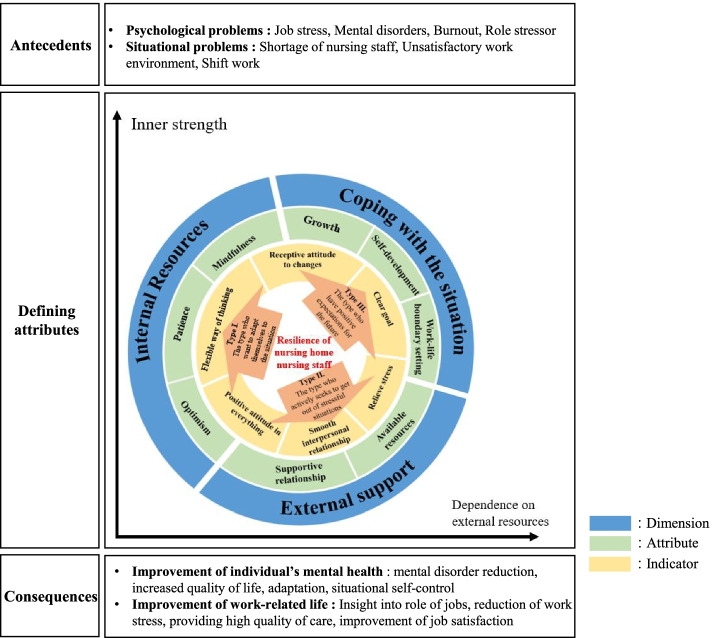
Conceptual structure of the resilience of nursing staffs in nursing homes

As a result of the final analysis, the attributes, dimensions, indicators, antecedents, consequences, related concepts, and types of resilience were identified (Table 2). All participants showed all the attributes and indicators of resilience examined in the final analysis, but in this study, the intensity of the attribute and indicator of each case was analyzed based on the interview contents, and the conceptual structure was schematized based on the results (Fig. 2).

**Table 2:** Dimension, attribute, and indicators of resilience of nursing home nursing staff

	Dimension	Attribute	CASE 1	CASE 2	CASE 3	CASE 4	CASE 5	CASE 6	CASE 7
Dimensions and attributes	Internal resources	Optimism	+++++	+++++	+++	+++++	+++	+++	+++
		Patience	+++++	+++	+++	+++	+++	+++++	+++
		Mindfulness	+++++	+	+++	+++++	+	+++++	+++++
	External support	Supportive relationship	+++	+++++	+	+++	+++	+++	+++++
		Available resource	+++	+++++	+	+	+++	+++	+++++
	Coping with the situation	Work-life boundary setting	+	+	+++++	+++	+++++	+++	+
		Self-development	+	+++	+++++	+	+++++	+++	+
		Growth	+	+++++	+++++	+	+++++	+++	+
Indicators	Relieve stress		+	+++	+++++	+	+++	+++++	+++++
	Flexible way of thinking		++++++	+++++	+++	+++++	+++	+++	+
	Smooth interpersonal relationship		+	+++++	+++	+++	+++	+++++	+++++
	Have a clear goal		+	+	+++++	+	+++++	+	+++
	Positive attitude in everything		++++++	+++++	+++	+++++	+++++	+++	+++
	Receptive attitude to challenges		+++	+++	+++++	+++	+++++	+++	+++

Intensity: +: Low; +++: Moderate; +++++: High

#### Attributes, dimensions, and indicators

The attributes, dimensions, and indicators of the resilience of NH nursing staff were analyzed while comparing the literature analysis and fieldwork stage results. That analysis and comparison confirmed optimism, patience, mindfulness, supportive relationships, available resources, work-life boundary setting, self-development, and growth as the final attributes of NH nursing staff resilience. The dimensions identified in the final analysis stage were internal resources, external support, and positive coping with difficult situations. As a result of comprehensively analyzing the indicators, NH nursing staff were classified into three types: those who want to adapt themselves to the situation, those who actively seek to cope with stressful situations, and those who have positive expectations for the future.

#### Antecedents

The antecedents of resilience can be divided into psychological and situational problems. The psychological problems found to be experienced by NH nursing staff were job stress, mental disorders, burnout, and role stressors. Their situational problems were traumatic situations, staff shortages, unsatisfactory work environments, shift work, and uncooperative patients and caregivers.

#### Consequences

The consequences of resilience can be broadly divided into two parts: improvement of individual nurse’s mental health and improvement of work-related life. The improvement of individual nurses’ mental health resulted in mental disorder reduction, increased quality of life, adaptation, and situational self-control. The improvement of their work-related lives led to developing insights into their roles as nurses, reducing work stress, providing a higher quality of care to their patients, and increased job satisfaction.

#### Related concepts

Through the theoretical and fieldwork stages, we were able to find both similar and contrary concepts. The ability to use judgment, make good decisions, and perceive change as beneficial when faced with the stressful challenges of life is called hardiness [44, 45]. Nurses with hardiness are more likely to be committed to the job, experience less burnout, and are less likely to give up or resign [46]. As such, hardiness and resilience have similar meanings as both reflect internal characteristics that buffer the negative effects of stress and play an important role in personal well-being and psychological sustainability [47]. However, hardiness develops early in life and is regarded as part of an individual’s personality traits and dispositions, and strength is also regarded as dispositional resilience [48]. Thus, hardiness has a similar but more limited meaning than resilience.

The contrary concept is burnout, which includes physical and emotional fatigue and stress due to stressful situations, which can result in an individual quitting work [49]. This concept includes both physical and psychological symptoms, and can lead to anger, irritability, and a negative attitude toward one’s job, finally resulting in resignation [50]. Burnout has a meaning contrary to that of resilience and is also viewed as an antecedent factor.

#### Types of nursing home nursing staff resilience

The types were categorized by CASEs (each participant) that appeared strongly in the indicator, and the strength of the attribute was also considered (Table 1). Based on the results of the analysis, the resilience of NH nursing staff was divided into three types. The three types are those who seek to adapt themselves to the situation, those who want to cope with a stressful situation, and those who have positive expectations for the future. The researchers found that when categorizing types, internal strength and dependence on external resources became important axes; hence, the types were categorized in three directions based on those two axes. It is possible to increase the clarity of the concept together with Wilson’s conceptual analysis method [22, 25]. Model cases that fully exhibit the attributes and indicators of the resilience concept is presented by type below.

##### Type I. those who seek to adapt themselves to the situation

This type is willing to accept the situation they are facing, maintains a positive attitude, and has a flexible mind. In addition, this type tries to overcome crises by adapting themselves to the situation and has a strong tendency to solve problems using their internal resources. This type of characteristic was well demonstrated in Cases 1, 2, and 4.

() : attribute; [ ] : indicator

*Case 1 is a nurse in her 40s who has been working in a NH for fifteen years. Case 1 well demonstrated her ability to overcome crises through her emotional stability but expressed that she felt most frustrated when caregivers did not trust the nurse and complained frequently. Meaningful statements from her include: "All the answers are in my mind. When I try to look at a situation in a positive way, it flows positively" (Optimism) [Positive attitude in everything]. "I work with elderly people, so it takes a lot of waiting. Difficult situations arise, but I don't respond to every situation. I don't care about things I can't control. Often the problem is solved by waiting" (Patience) [Flexible way of thinking]. “As I think about the goals of my life as a nurse, I try to look objectively at what is bothering me. I meditate every morning and try to look at it objectively, repeatedly reminding myself of the things that are bothering me” (Mindfulness) [Having a clear goal].*

##### Type II. Those who actively seek to cope with stressful situations

This type sought to cope with stressful situation by using the resources available to them. This type tried to reduce stress by using one’s own strategies and was actively helped by relying on close relationships. They tried to solve the problems they faced by using these active methods. Cases 6 and 7 demonstrated these characteristics well.

*Case 6 is a nursing assistant in her 30s who has been working at a NH for eight years. She had a difficult time receiving constant complaints from a resident's family due to the recent resident's pressure sores. But she tried to cope with the difficult situation quickly. Meaningful statements from her include: "I received a lot of comfort from my colleagues. All my colleagues enjoy good relationships and support each other in difficult times. We always take good care of each other and get along well" (Supportive relationships) [Smooth interpersonal relationships]. "I have a lot of people to get help from. The manager of this NH is always interested in us and gives us vacation time when we look tired” (Available resources). “On my days off, I usually travel" (Work-life boundary setting) [Relieving stress].*

##### Type III. Those who hold positive expectations for the future

This type focuses on work by clarifying the scope of their work and striving to develop themselves. Through these efforts, they seek to grow step by step and to cultivate positive expectations for the future. Positive expectations for the future are the source of their strength to overcome crises. Cases 3 and 5 showed the characteristics of this type well.

*Case 3 was a nursing assistant in her 30s who had worked for five years in* a NH*. She explained that the stress at her job was bothering her, even when she returned home from her job. Case 3 exemplifies overcoming her crisis by coping with her situation. She told us the following: "I couldn't stand being bothered at home because of the stress of work. So, when I got home, I put my phone on silent and tried not to think about work. I instead tried to get enough rest, meet friends, and forget about work” (Setting the boundaries of work) [Relieving stress]. I felt that I had to understand the elderly well to be able to do my job effectively, so I voluntarily searched for education related to elderly care, studied it, and tried to accumulate knowledge. In the future, I want to become a senior nursing specialist and provide professional nursing care” (Self-development, Growth) [Receptive attitude to challenges, Having a clear goal].*

#### Conceptual structure

From the derived results, the relationship between dimension, attribute, and type could be expressed as shown in Fig. 2, focusing on the characteristics strongly expressed in the concept. Each attribute belonging to the three types is organically connected to constitute the concept of resilience. In the case of type 1, the attribute belonging to the internal resources dimension was emphasized, and in the case of type 2, the attribute belonging to external support was prominent. In the case of type 3, it was found that many attributes of coping with the situation were expressed. Considering both indicator and attribute, the type could be expressed with inner strength and dependence on external resources as axes.

## Discussion

This study defined the concept of resilience of NH nursing staff and explored the conceptual characteristics. Through this study, it was found that the resilience of NH nursing staff has a complex conceptual structure. This concept consists of three dimensions, internal resources, external support, and coping with the situation; includes eight attributes, optimism, patience, mindfulness, available resources, supportive relationship, work-life boundary setting, self-development, and growth; and is divided into three types based on two axes, inner strength and dependence on external resources.: those who seek to adapt themselves to the situation, those who actively seek to cope with stressful situations, those who hold positive expectations for the future.

### The dimension of internal resources

This dimension can be considered a personal internal characteristic of nursing staff. Compared with previous studies which examined the concept of resilience, it was confirmed that the results of this study emphasized the attributes of patience and mindfulness. According to previous studies, nurses, and nursing assistants in NHs for the elderly feel unwanted emotions in the process of caring for the elderly, and patience is one of the ways they cope with these potentially distressing situations [51]. The patience and mindfulness uncovered in this study are similarly regarded as responses to unwanted emotions experienced in the process of caring for the elderly. To help improve their resilience, research on unwanted and distressful situations encountered by NH nursing staff should be undertaken. In addition, it can be suggested that designing interventions that could develop the internal strengths and resources of NH nursing staff members would increase their resilience.

### The dimension of external support

In this dimension, the participants showed an attitude of actively seeking help through available resources and supportive relationships when attempting to overcome crises. Previous studies on the concept of nurses’ resilience identified seeking comfort through positive interpersonal relationships as well as help to solve their difficulties as strategies for overcoming adversities [52]. Therefore, in improving the resilience of NH nursing staff, including nurses, community formation such as creating self-help groups that can encourage and maintain close relationships with colleagues can help build resilience for overcoming difficult situations.

### The dimension of coping with difficult situations

This dimension was best typified by those with positive expectations for the future, who showed the most attributes of this dimension. This dimension reflects the characteristics of clearly setting boundaries between work and life, along with self-development and growth. In previous studies, work-life interference was found to significantly increase burnout, contribute to increasing turnover intention, and lower career satisfaction [53]. For shift workers, managers’ interventions for sustaining a healthy work-life balance were found to improve workers’ satisfaction and well-being [54]. Therefore, efforts are required to improve NH working environments by introducing programs at the NH facility and manager levels that can maintain a healthy work-life balance and reduce work-life interference. In addition, previous studies have shown that people tend to overcome crises and grow by concentrating on their personal time and making good use of their leisure time when experiencing traumatic situations [55]. It was also found that, for nurses, developing resilience through self-development is helpful for professional development [56]. Therefore, it is considered that support at the national level as well as at the facility level is necessary to improve working conditions at NHs and enable nursing staffs to invest time in self-development.

This study was conducted by focusing on participants with resilience attributes determined through an in-depth literature review. Resilience includes all those attributes, and the classification of types based on attribute intensity can be used as important evidence when developing interventions in the future.

The hybrid model is a methodology that synthesizes the search for theoretical knowledge and the search for empirical knowledge based on the meaning and measurement of concepts [22], essential and basic research for concept development and theory development in practice. Therefore, based on this study, we propose developing a tool to measure the resilience of NH nursing staffs. Such a tool could be used to collect basic data for NH resilience intervention studies and might serve as a catalyst for future studies.

This study is meaningful in that it suggests a research direction for related research to be carried out in the future by trying to clarify and structure the concept of resilience of the complex NH nursing staff.

### Limitations

Since the participants were recruited based on the attributes revealed in the theoretical stage and the data were collected via electronic communication due to the COVID-19 pandemic situation, contrary and borderline cases could not be observed. In the future, it will be necessary to conduct research that can be compared with the results of this study by collecting contrary and borderline cases using various participant approaches.

## Conclusion

The NH nursing staff participants demonstrated their resilience through attempting to positively cope with difficult situations by using internal resources and external support. The conceptual characteristics of the resilience of NH nursing staff revealed in this study suggested a direction for future studies that could improve the resilience of such caregivers. Based on this, it is expected that meaningful research on how to improve the resilience of NH nursing staff will be conducted in the future.

## Data Availability

All data generated or analyzed during this study is included in this published article.
